# When Homing Endonuclease Meets Transposon: The OMEGA System

**DOI:** 10.3390/biology15171491

**Published:** 2026-09-02

**Authors:** Kenji K. Kojima

**Affiliations:** 1 Genetic Information Research Institute, East Palo Alto, CA 94303, USA; kojimakk@arizona.edu; 2 R. Ken Coit College of Pharmacy, University of Arizona, Tucson, AZ 85721, USA

**Keywords:** homing endonuclease, the OMEGA system, TnpB, Fanzor, intron, intein, sequence-specificity, Cas9, Cas12, transposon

## Abstract

Sequence-specific DNA endonucleases have made significant contributions to biology, biotechnology, and medicine; restriction enzymes and homing endonucleases are among classic examples. The demonstration of programmable genome editing using Cas9 in the CRISPR-Cas system, in which the target DNA sequence is recognized by base pairing with a guide RNA, revolutionized the field of genome engineering, making target selection more flexible and convenient. The OMEGA (Obligate Mobile Element-Guided Activity) system, considered a precursor to Cas12, and likely to Cas9, in the CRISPR-Cas system, is an RNA-guided DNA endonuclease composed of a TnpB, IscB, IsrB, or Fanzor protein, and a structural RNA designated reRNA or ωRNA. The OMEGA system is present in the three domains of life as an auxiliary component of transposons. The OMEGA system cuts DNA in an allele from which a transposon is excised and triggers recombination to reinstate the transposon at the same position. This “transposon restorative homing” redefines the OMEGA system as a homing endonuclease. In this review, the selfish aspects of the OMEGA system are discussed in the historical context of homing endonuclease research.

## Simple Summary

The CRISPR-Cas9 and CRISPR-Cas12 systems recognize target DNA through base pairing with their guide RNAs, revolutionarily simplifying the design of DNA endonucleases with novel target-sequence specificity for genome editing and gene therapy, compared with endonucleases that rely on protein-based target recognition. The OMEGA (Obligate Mobile Element-Guided Activity) system, considered a precursor to Cas12, and likely to Cas9, in the CRISPR-Cas system, is widely present in the three domains of life as an auxiliary component of transposons. Recent studies have shown that the OMEGA system cleaves the site from which a transposon was excised, thereby inducing transposon restoration via gene conversion. It is now evident that the OMEGA system acts selfishly as a homing endonuclease. This article will focus on the selfish behavior of classical homing endonucleases and the OMEGA system, and discuss how it has evolved, been maintained, and adapted to new genomic environments.

## 1. Introduction

A selfish genetic element (SGE) is defined as an element that has characteristics that enhance its own transmission relative to the rest of an individual’s genome but neutral or detrimental to the organism as a whole [1]. Genetic conflict occurs when an increase in transmission of one element due to its phenotypic effects causes a decrease in the transmission of other elements. The characteristics of SGEs can be distilled into two types: over-replication and transmission distortion [2].

Transposons (transposable elements, TEs) represent the former group: over-replicators that tend to replicate more than the rest of the genome. Transposons can (copy and) move to new locations within the genome. Transposons are traditionally classified into two categories: Class I (retrotransposons) and Class II (DNA transposons) [3,4]. Retrotransposons replicate themselves through transcription and reverse transcription. Reverse transcription can be coupled, in the cases of non-long terminal repeat (non-LTR) retrotransposons and *Penelope*-like elements, or uncoupled with integration, in the cases of LTR retrotransposons (including retroviruses) and YR (or *DIRS*) retrotransposons. DNA transposons cut or copy their own DNA and integrate it at another location. Transposition of DNA transposons is catalyzed by several distinct enzymes called transposases, integrases, and recombinases.

The other strategy SGEs take is transmission distortion, in which an element is inherited by offspring at a higher rate than its competitor (allele). For example, the toxin–antitoxin system (TA system or toxin–antidote system) kills the offspring cells if they do not maintain the TA system [5]. The TA systems are often found in plasmids or transposons. The toxin generally persists longer than its antitoxin, which ensures that daughter cells die if the TA system is lost. Gene converters are a special class of SGEs that preferentially insert themselves into homologous, uninserted sites in the genome. The most famous group of gene converters is homing endonuclease (HE) genes, which are found in all three domains of life.

## 2. Homing Endonucleases

Sequence-specific endonucleases are the key contributors to biotechnology and genome engineering. Restriction enzymes—strictly sequence-specific endonucleases found in restriction-modification (RM) systems in prokaryotes—enabled us to predictably recombine DNA [6]. The RM system is a type of prokaryotic defense system that prevents phage invasion, but it also functions as a selfish entity that forces the host to keep itself through post-segregational killing, very similarly to the TA system [7]. The RM system is now classified into four types (I, II, III, and IV), with Type II restriction enzymes being the most suitable for application as they cleave DNA at a specific position in the recognized target DNA sequence [6]. Since the RM system needs to distinguish modified DNA bases (self) from unmodified bases (non-self), restriction enzymes recognize DNA bases very strictly. Type II restriction enzymes such as EcoRI and BamHI usually recognize 4 to 8 bp sequences; these short recognition sequences provide enough cleavage sites in foreign DNA (usually phages and plasmids) to inactivate them.

HEs, even though they are also sequence-specific endonucleases, show quite distinct characteristics compared to restriction enzymes; they recognize relatively long stretches of DNA (14 to over 40 bp). The length of recognized DNA sequences restricts the cleavage site, usually to a single site in the entire genome. HEs simultaneously tolerate sequence polymorphisms at poorly conserved bases in the host target site.

HEs can be grouped into several structural families, which are defined by conserved catalytic motifs and protein folds. The families are LAGLIDADG, GIY–YIG, HNH, His–Cys box, PD-(D/E)xK, EDxHD, and DHHRN [8,9,10]. Among them, HNH and His-Cys box show similar structures called ββα-metal, and PD-(D/E)xK and EDxHD show divergent but related structures. Except for LAGLIDADG, these structures are also found in non-HEs. Despite sharing the same general biological role, these families evolved distinct structural solutions for DNA recognition and cleavage. HEs are found in phages, bacteria, archaea, microbial eukaryotes, organellar genomes, and viruses, and are often associated with self-splicing introns, archaeal introns, or inteins.

The LAGLIDADG family is the best characterized structurally and includes both homodimeric enzymes with one catalytic motif per subunit and monomeric enzymes containing two duplicated motifs within a single polypeptide [11]. These proteins typically recognize long DNA targets through extended α-helical interfaces containing the LAGLIDADG motif and often generate staggered double-strand breaks. The protein employs many weakly coupled contacts, which allows specificity with sequence tolerance at each position.

Thanks to their long target recognition, HEs are promising tools for many applications, including gene correction, targeted mutagenesis, and therapeutic genome engineering [11]. Considerable efforts have been devoted to engineering HEs to target novel DNA sequences beyond their natural specificities. Early studies focused on members of the LAGLIDADG family, such as I-CreI and I-MsoI, whose modular DNA-binding interfaces enabled rational redesign through structure-guided mutagenesis [12,13,14]. Simultaneously, chimeric endonucleases generated by domain swapping expanded the range of accessible target sites [15,16]. Over the past two decades, researchers have generated engineered variants capable of recognizing dozens to hundreds of novel target sequences, including therapeutically relevant loci in human cells [11,17]. These studies demonstrated that HEs can serve as genome-editing reagents.

While thousands of target sequences of natural and artificial HEs have been reported collectively, retargeting success has remained difficult and inflexible because of the highly interconnected nature of protein–DNA interactions in HEs and the limited variety of natural target sequences [11,18,19]. Individual amino acid substitutions often alter neighboring contacts, DNA bending, catalytic geometry, or overall protein stability. Many new targets of artificial HEs differ by only a few nucleotide substitutions from their parent recognition sites. Since the development of CRISPR-Cas systems-based DNA cleavage with programmable guide RNAs [20], they have rapidly superseded engineered HEs for most genome editing applications.

## 3. Selfish Behaviors of HE Genes

### 3.1. Intron Homing

The HE gene (HEG) was first discovered in studies on the significant bias in the inheritance of the ω+ rRNA gene markers in yeast mitochondria during crossing [21]. In gametes, maternal and paternal mitochondrial genomes can recombine. Mitochondria in the offspring showed the ω+ alleles at frequencies much higher than the 50% predicted by Mendel’s laws. This super-Mendelian inheritance was accomplished by the endonuclease activity encoded within the large ribosomal RNA gene at the ω+ allele. The DNA double-strand break induced by the endonuclease at a cognate (ω−) allele lacking the ω+ gene is repaired by gene conversion using the ω+ allele as the template.

Homing is the process through which an SGE promotes its own inheritance by introducing a site-specific DNA break at a homologous target site lacking the element, thereby stimulating gene conversion that copies the element into the recipient locus. This process converts an element-negative allele into an element-positive allele. Consequences include non-Mendelian transmission and preferential inheritance of SGEs.

**Figure 1:**
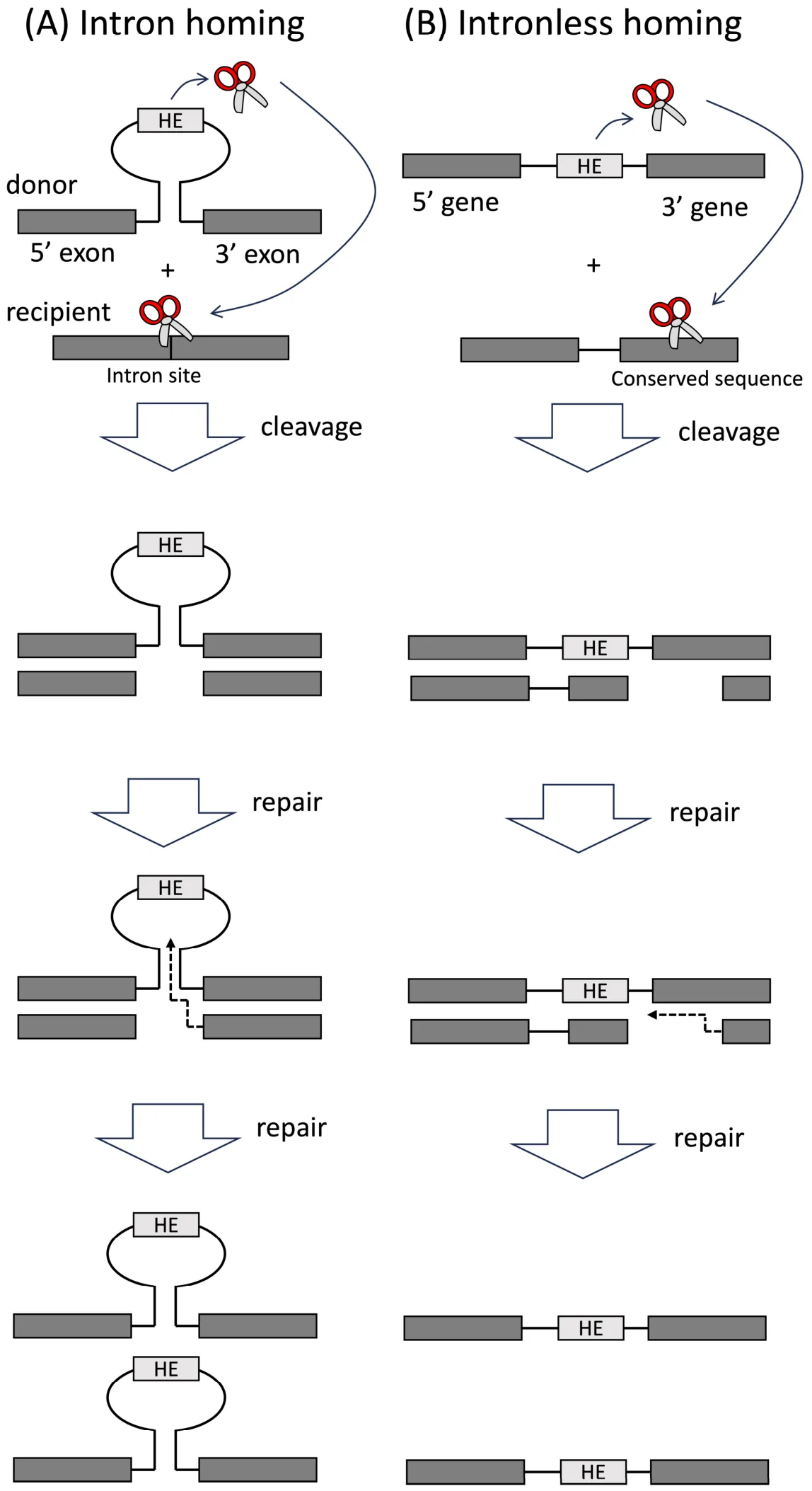
Two strategies for maintaining and spreading HE genes. (**A**) Intron-encoded HE is translated and cleaves the empty locus of the recipient allele. The DNA double-strand break is repaired using the donor allele as the template. (**B**) Free-standing HE is translated and cleaves a conserved sequence inside a gene. The DNA double-strand break is repaired using the donor allele as the template. Because sequence similarity between the donor and recipient alleles is restricted mainly to genes, a long sequence near the recipient cleavage site is converted.

HEGs are most commonly found in group I self-splicing introns [18]. HEs encoded in a self-splicing intron cleave an intronless allele near the intron insertion site, and the DNA break is repaired by gene conversion with the intron-containing allele used as the template (Figure 1A). In sexually reproducing organisms, HEGs can spread in the population, converting the HEG- allele into the HEG+ allele. Self-splicing minimizes the impact of HEG insertions on fitness. Introns are usually inserted at highly conserved sequences inside essential genes. It prevents the elimination of introns in the population as only the precise removal of introns can restore the original intronless genes. As HE cleaves DNA by recognizing the sequence surrounding the intron insertion site, the presence of introns protects DNA from cleavage.

The presence of HEGs inside group I introns is evolutionarily unstable. HEGs need intronless alleles to spread in the population. Once the intron is fixed in the entire population, there is no target sequence for HEs, and as a result, HEGs accumulate mutations. The classic model explains the maintenance of HEGs by horizontal transfer across species or population [22,23], but their persistence without horizontal transfer is possible, depending on the cost of nonfunctional HEGs and the frequency of homing [24].

Although less common than group I introns, the LAGLIDADG family of HEGs in group II self-splicing introns has evolved multiple times across diverse organisms and viruses [25,26,27]. Archaeal bulge–helix–bulge introns also harbor HEGs [28]. HEGs are also found in inteins [29]. An intein can be defined as a self-splicing protein element that excises itself and ligates the surrounding protein segments (exteins) to form a mature, functional protein. Inteins are most abundant in archaea, where many essential genes contain them [30].

Eukaryotes, especially multicellular eukaryotes, depend more on non-homologous end joining rather than homologous recombination to repair double-strand breaks [31,32], which makes homing less adaptive.

### 3.2. Intronless Homing

HEGs are also observed as free-standing genes surrounded by conserved genes. Free-standing HEGs have been most extensively documented in bacteriophages [33]. In T-even phages such as phage T4, free-standing HEGs, including Seg and Mob, promote the mobility of adjacent genomic segments to related phages (such as T2) during co-infection [34,35] (Figure 1B). This phenomenon was originally reported for intron-encoded HEGs in phages as “coconversion”, the co-transfer of flanking phage genes alongside a mobile genetic element (in this case, an HEG) during genetic recombination, or “localized marker exclusion”, where specific genetic markers from one phage are drastically underrepresented in the progeny [36,37,38].

The HE preferentially cleaves the recipient phage genome rather than the donor phage genome [34]. The sequences surrounding the cleavage site are almost identical between these phage genomes (31 bp identical sequence in the case of SegF); therefore, the HE recognizes a longer stretch of DNA to distinguish the two phage genomes. This is an additional constraint for intronless homing compared with intron homing, where the target sequence with or without an intron is easily distinguished. The double-strand break is repaired using the donor genome as the template. Two well-conserved genes between the two types of phage genomes sandwich the HEG. The cleavage of the recipient phage genome by the HE results in the spread of the HEG into the recipient phage genome. Such behavior of free-standing HEGs drives the spread of neighboring DNA modules. When an HEG and a self-splicing intron are in close proximity and target the same sequence, they can collaboratively spread together [39,40]. Such “collaborative homing” may represent the transitional stage between free-standing HEGs and intron-encoded HEGs.

As coconversion/localized marker exclusion is the transfer of genetic information from one phage species to another, one expected outcome is the conserved cluster of genes including an HEG across diverse phage genomes [41]. Such “HEG-islands” are not expected to have discrete boundaries.

When two phage sequences are too divergent for effective gene conversion but the target sites of the HEs still show marked similarity, the HEG provides a competitive advantage to the host phage by killing its competitors [42]. Homing and killing competitive phages simultaneously likely contribute to the proliferation of HEGs.

## 4. Cas9– and Cas12—RNA-Guided Endonucleases in Class 2 CRISPR-Cas Systems

**Figure 2:**
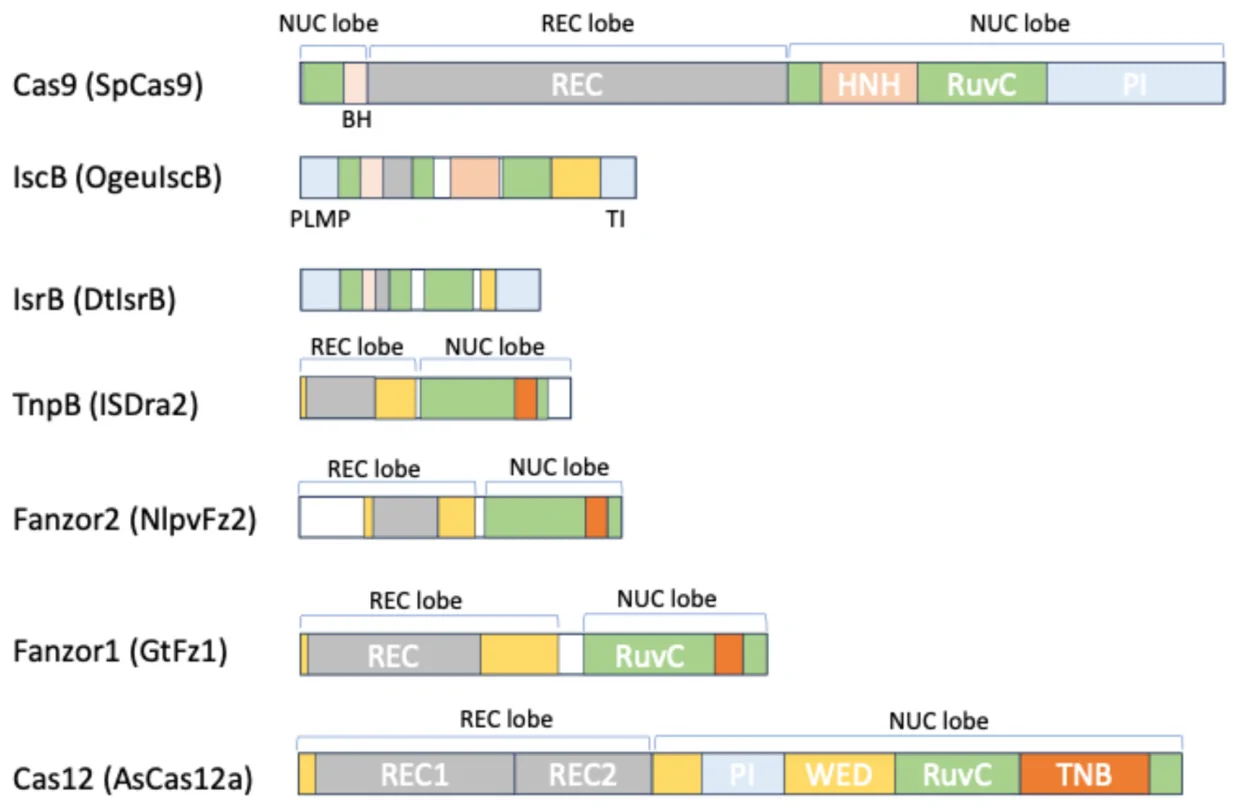
Schematic structures of Cas9, Cas12, and the effector proteins in the OMEGA system. Domain structures are based on [48,49,50,51,52,53]. The RuvC domain (green) is divided into subdomains.

The recent and remarkable additions to the list of sequence-specific endonucleases are endonucleases that originated from prokaryotic immune systems. Cas9 and Cas12 are distinct but related effector proteins responsible for the target DNA cleavage in the CRISPR-Cas system [20,43,44]. Both show a bilobed architecture, comprising REC and NUC lobes [45,46,47] (Figure 2). Cas9 contains an HNH nuclease domain intervening between its RuvC nuclease domain, while Cas12 lacks an HNH nuclease. The RuvC domain is split into three discontinuous subdomains (RuvC-I to III), and three catalytic residues (D, E, and D) are distributed among the subdomains.

The CRISPR-Cas system is an adaptive immune system found in bacteria and archaea that protects cells from genetic invaders such as phages and plasmids [54,55,56]. The system consists of clustered regularly interspaced short palindromic repeat (CRISPR) arrays—genomic loci containing short repeated sequences separated by “spacers” derived from past invaders—and CRISPR-associated (Cas) proteins that process guide RNAs and cleave foreign nucleic acids. The CRISPR-Cas systems are highly diverse and are classified into two major classes and multiple types/subtypes [57]. Class 1 systems use multi-protein effector complexes, whereas Class 2 systems use a single large effector protein. Cas9 is a component of a Class 2 Type II CRISPR-Cas system, and Cas12 is part of a Class 2 Type V system.

RNA-guided targeting in Cas9 and Cas12 systems allows for programmable recognition of nearly any sequence adjacent to a protospacer adjacent motif (PAM), making CRISPR tools extraordinarily versatile. Since the demonstration of programmable genome editing with Cas9 [20], CRISPR technologies have transformed molecular biology, enabling genome editing, gene regulation, epigenome modification, diagnostics, and therapeutic development.

The emergence of the CRISPR-Cas9 system transformed engineered gene drives from a technically challenging concept into a practical and widely accessible technology. The conceptual basis of gene drive is tightly linked with the selfish behavior of HEGs [58]. HEGs could be used to drive linked genetic cargo through a population even if the cargo imposes a fitness cost. The expected features for HEGs in gene drive were to recognize and cut a sequence in the middle of an essential gene whose knockout is recessive. Natural and retargeted I-SceI were applied for gene drive in *Drosophila melanogaster* and *Anopheles gambiae* in the laboratory, demonstrating the potential of HEGs for gene drive while also revealing their limitations [59,60]. These systems could, in principle, bias inheritance but they were difficult to redesign for new genomic targets. Even early applications of an engineered CRISPR-Cas9 system for gene drive showed high inheritance efficiency in *D. melanogaster* [61] and *A. gambiae* [62] thanks to more flexible target gene selection.

It is now obvious that the engineered CRISPR-Cas9/Cas12 system functions as an alternative to HEGs. But what does this resemblance mean in the light of evolution? The characterization of the OMEGA (Obligate Mobile Element-Guided Activity) system has illuminated the connection between HEGs and the CRISPR-Cas9/Cas12 system.

## 5. The OMEGA (Obligate Mobile Element-Guided Activity) System—A Composite Homing Endonuclease

### 5.1. The IS200/IS605 Family—A Compact Insertion Sequence with a HUH Nuclease/Y1 Transposase Gene

**Figure 3:**
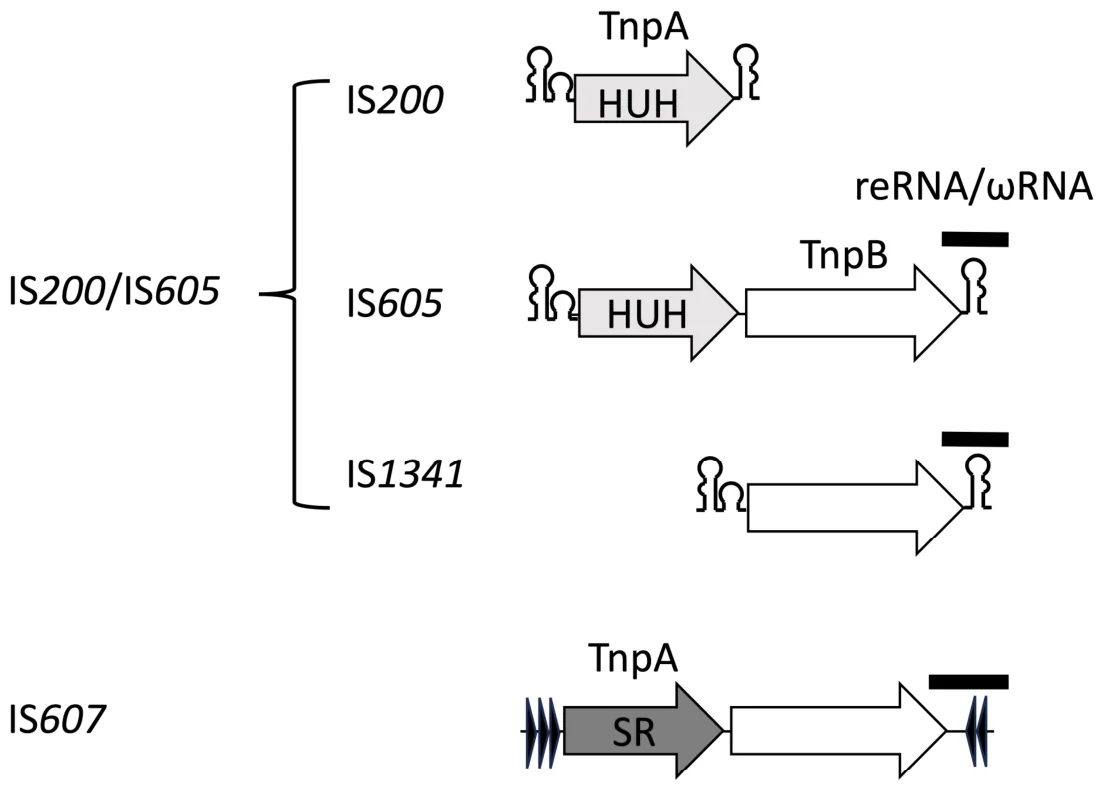
Schematic structures of the IS*200*/IS*605* family and IS*607* family of ISs. HUH, HUH nuclease; SR, serine recombinase. The RNA secondary structure cartoons at both ends of IS*200*/IS*605* indicate the small hairpin structures recognized by TnpA. Triangles in IS*607* represent repeat units.

The IS*200*/IS*605* family comprises small insertion sequences (ISs) that transpose via a single-stranded DNA (ssDNA) “peel-and-paste” mechanism catalyzed by a small transposase (TnpA). The first member of this family, IS*200*, was identified in 1983 from *Salmonella* [63]. IS*608* (IS*Hp608*) from *Helicobacter pylori* and IS*Dra2* from *Deinococcus radiodurans* have been studied in detail [64,65,66,67,68,69]. Based on the coding ability of two proteins, TnpA and TnpB, they are divided into three major groups [70]. IS*200* encodes a single protein, TnpA, while IS*605* encodes two proteins, TnpA and TnpB (Figure 3). TnpB is not required for transposition [64]. IS*1341* only encodes TnpB.

The IS*200*/IS*605* family does not possess terminal inverted repeats (TIRs) or generate target site duplications (TSDs). Instead, the left and right ends include sequences that form DNA hairpins. Members of the IS*200*/IS*605* family show slightly different target sequence specificity: a tetranucleotide TTAC in the case of IS*608*, TTTAA or TTAAC in the case of IS*605*, and TTGAT in the case of IS*Dra2*, which are adjacent to the 5′ terminus [69,71].

During transposition, a specific strand of the transposon is excised to form an extrachromosomal single-stranded transposon circle [64,65,66]. This inserts into a single-stranded target. TnpA recognizes small hairpin structures in a strand-specific manner and catalyzes single-strand cleavage at both ends. It also requires a conserved Y residue downstream of the HUH motif. The two Hs coordinate a divalent metal ion cofactor required for catalysis, while the Y acts as a nucleophile and forms transient 5′ phosphotyrosine enzyme–DNA intermediates.

A study of multicopy Cas9 homologs found a group of distant relatives of the IS*200*/IS*605* family [72]. They were denoted ISC (Insertion Sequence encoding Cas9 homolog). ISC resembles IS*200*/IS*605* in terminal structures and protein-coding capacities, but its Cas9 homolog (IscB) is more similar to Cas9 than TnpB of IS*605*. ISC was divided into two groups based on the presence of an HNH endonuclease inserted in the RuvC-like domain of IscB (ISC2) or its absence (ISC1) (Figure 2). The Cas9-like protein lacking an HNH endonuclease was later renamed IsrB (Insertion Sequence RuvC-like OrfB) [49] (Figure 2). Though most ISC members do not encode additional proteins other than IscB/IsrB, some encode an HUH nuclease homologous to TnpA in IS*200*/IS*605*; such families were designated ISC2Y. Independently reported HEARO (HNH endonuclease-associated RNA and ORF) [73] was revealed to be phylogenetically clustered together with IscB [74].

### 5.2. The IS607 Family—A Unique Insertion Sequence with a Serine Recombinase Gene

The IS*607* family is a group of unusual ISs that transpose using serine recombinase-type transposases, rather than the more common DDE transposases or tyrosine recombinases [70] (Figure 3). The IS*607* family neither has TIRs nor generates TSDs. Most IS*607* family members terminate in GG. The IS*607* family was first described in the human pathogens *Mycobacterium tuberculosis* (IS*1535*) and *Helicobacter pylori* (IS*607*) [75,76]. It is now known that the IS*607* family is distributed across diverse bacteria and archaea [77,78]. IS*607*-like sequences are also observed in eukaryotes and viruses infecting eukaryotes [79,80,81,82].

The IS*607* family encodes two proteins. One is a serine recombinase (TnpA) responsible for transposition [83]. The other is TnpB, which is not essential for transposition and is homologous to the TnpB observed in the IS*200*/IS*605* family. The involvement of circular intermediates of double-stranded extrachromosomal DNA was experimentally validated [84]. The IS*607* family is strictly integrated into or downstream of a GG dinucleotide.

### 5.3. TnpB, IscB, IsrB—Three Homologous Protein Groups That Associate with Guide RNA to Form RNA-Guided DNA Endonucleases

As mentioned above, three protein families, TnpB, IscB, and IsrB, encoded by the IS*200*/IS*605* family and the IS*607* family, show significant sequence similarity to Cas9 and Cas12 in the CRISPR-Cas system [72] (Figure 2). Indeed, TnpB was revealed to be an RNA-guided DNA endonuclease [71]. TnpB from IS*Dra2* (IS*200*/IS*605* family) is bound to an RNA (right end element RNA, reRNA) which is composed of the 3′ region of IS*Dra2* and around 16 nt sequence derived from the 3′ flanking genomic DNA (see Figure 3, IS*605*). The TnpB-reRNA complex cleaves a DNA sequence between the transposon-associated motif (TAM), which is TTGAT and is the identical sequence seen at the 5′ flanking of IS*Dra2*, and the sequence complementary to reRNA. Other IS*605* family members also show transcription of the 3′ part of the TnpB gene to the right end of the transposon; the transcript was named ωRNA and corresponds to reRNA [49]. IscB is bound to the ωRNA, which is transcribed from the 5′ side of the IscB gene, and also shows RNA-guided DNA cleavage, similar to TnpB [49]. Consistent with the lack of an HNH domain in IsrB, IsrB-ωRNA ribonucleoprotein nicks one DNA strand in a guide- and TAM-specific manner [49]. The ωRNA associated with IscB was previously reported as HEARO in the search for highly structured RNA [73,74]. RAGATH-18 (RNAs Associated with Genes Associated with Twister and Hammerhead 18) RNA, found in the same study, was revealed to also interact with TnpB of the IS*607* family to form an RNA-guided endonuclease [73,85]. TAM sequences of most IS*607* TnpBs are AGGAG or GAGGG.

IscB and IsrB are both much more compact than Cas9 due to the lack of the REC lobe, which is responsible for the target DNA recognition in Cas9 [45,86] (Figure 2). Instead, IscB and IsrB rely on their larger ωRNA scaffold to facilitate target selection [51,52]. On the other hand, TnpB contains a REC lobe similar to that of Cas12 [48,87]. TnpB of both the IS*200*/IS*605* family and the IS*607* family uses its REC lobe and RuvC domain to recognize a relatively short heteroduplex of guide RNA and target DNA [48,85,87].

**Figure 4:**
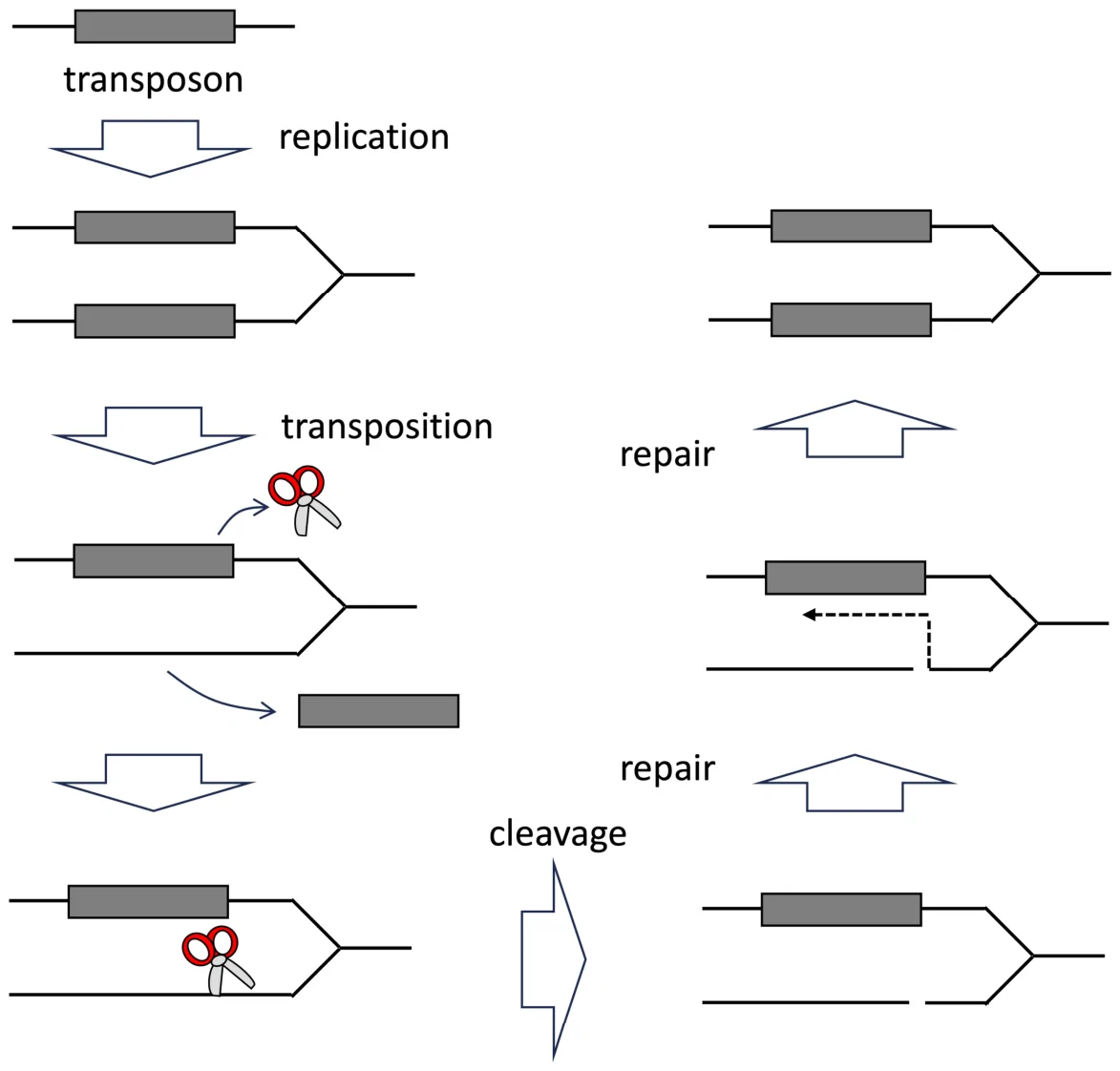
Transposon restorative homing. A transposon is excised from one chromatid during chromosome replication, and the transposon-encoded HE cleaves the empty site from which the transposon was excised. The DNA double-strand break is repaired using the chromatid with a transposon insertion through gene conversion, restoring the excised transposon.

The RNA-directed endonuclease activity of TnpB and IscB contributes to the maintenance of transposons [71,88] (Figure 4). During transposition, TnpA excises the IS*200*/IS*605* family of IS elements as a single-stranded DNA intermediate and inserts it into a new genomic site [88]. Although excision occurs on only one strand, removal of both strands has been experimentally validated; it remains to be determined how the second strand of the transposon is removed after the first DNA strand is transposed. This process leaves behind donor chromosomes that no longer contain the transposon. The insertion efficiency is much lower than the excision frequency, leading to the loss of transposon from the population. TnpB associates with an ωRNA and recognizes the empty site sequence on the donor chromatid. By cleaving DNA that lacks the transposon, TnpB stimulates gene conversion using the transposon-containing chromatid as the template, thereby restoring the transposon at the original locus. This “restorative homing” activity increases the retention and inheritance of the transposon within the population and counteracts losses caused by transposon excision. Restorative homing is also observed in the IS*607* family [84], thereby generalizing the concept to other transposons.

A canonical homing ability of TnpB has also been suggested [89]. The presence of IS*605* on a plasmid is detrimental to plasmid competition within cells. Nevertheless, IS*605* spreads to other plasmids that originally lacked the IS insertion and is fixed in the population more effectively than expected given its frequency. Catalytic inactivation of TnpA did not greatly affect the fixation of the IS*605*+ plasmid, while the inactivation of TnpB eliminated the advantage. This supports the function of TnpB as a gene drive agent of IS*605*.

The cleavage site by these endonucleases is determined by the sequence complementarity against the guide ωRNA and its 5′ adjacent TAM sequence. The ωRNA contains a guide sequence derived from the sequence adjacent to the transposon end. Consequently, the guide RNA is naturally complementary to the sequence that appears after excision. To cleave the empty donor sequence after transposon excision, the transposon must be inserted at the 3′ side of a sequence that fulfills the TAM requirement. The IS*200*/IS*605* family and the IS*607* family show distinctive 5′ target preferences: the IS*200*/IS*605* family is inserted downstream of AT-rich sequences [69] while the IS*607* family is inserted downstream of G-rich sequences [75]. These target preferences align well with the sequence preference in TAMs, reflecting the co-evolution between TnpA (an HUH nuclease for the IS*200*/IS*605* family and a serine recombinase for the IS*607* family) and TnpB. The recognition of TAMs via protein–RNA interactions differs between the two types of TnpB [48,85,87].

### 5.4. IStrons—Triple-Structured Selfish Genetic Elements

**Figure 5:**
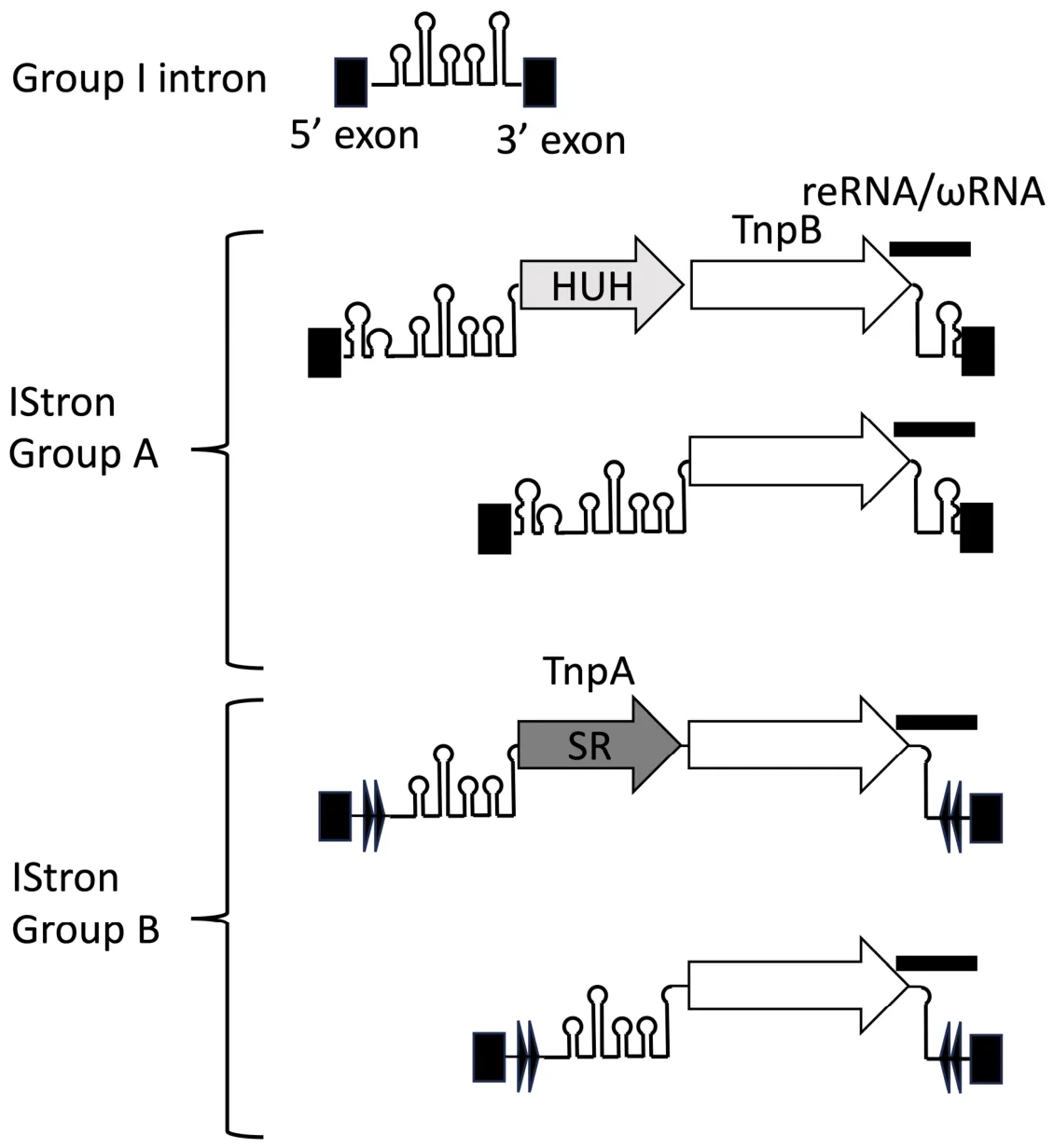
Schematic structures of group I intron and IStrons. HUH, HUH nuclease; SR, serine recombinase. The RNA secondary structure cartoons of group I introns and IS*200*/IS*605* are shown. Triangles in group B IStrons represent repeat units. reRNA/ωRNA extends beyond the 3′ end of the intron by ~15 nt.

IStrons were first identified in *Clostridium difficile* as the fusion of a group I self-splicing intron with an IS*200*/IS*605* family IS [90]. A related element was found in chromosomes of the *Bacillus cereus* group [91]. IStrons are highly variable among strains and are observed in unrelated protein-coding genes, indicating their transposition. Subsequent bioinformatics analysis revealed two types of IStrons, distinct in the terminal sequences and encoded proteins [92]. Group A corresponds to the fusion between a group I intron and an IS*200*/IS*605* family of ISs, while Group B corresponds to the fusion between a group I intron and an IS*607* family of ISs (Figure 5). In both types of IStrons, although the disruption of TnpA, or both TnpA and TnpB, is often observed, the disruption of TnpB only has not been observed. The intron component of IStrons varies, but all are classified as IA2.

Group A IStrons are inserted downstream of the pentanucleotide TTGAT, ATTAT, or TTTAT, which is consistent with the target preference of the IS*200*/IS*605* family, and their 3′ terminus is TCAG. The last nucleotide of the 5′ exon is T, like almost all other group I self-splicing introns. In group I introns, the U at the 5′ exon–intron junction is part of the U-G base pairing that determines the 5′ splice site. Group B IStrons are inserted after GG (AGGG, TGGG or GAGG) and terminate with CGG at the 3′ end. These sequence preferences are consistent with those of the IS*607* family. However, this results in the unusual last nucleotide at the 5′ exon adjacent to Group B IStrons being a G, not T. The G at the 5′ exon–intron junction is paired with C inside of Group B IStrons. This boundary was confirmed experimentally in vivo. Phylogenetic analysis of introns and proteins suggests two independent origins of IStrons, corresponding to Groups A and B.

IStrons are a composite system that has three functions: self-splicing, transposition, and homing. Self-splicing requires the functional ribozyme, mostly originating from the 5′ untranslated region of IStrons. TnpA (either via HUH nuclease or serine recombinase function) catalyzes the DNA excision and integration by recognizing the sequences around the 5′ and 3′ junctions of IStrons. Finally, TnpB catalyzes the DNA cleavage through the sequence complementarity between the target DNA and ωRNA, which is composed of the 3′ region of IStrons and the 3′ flanking sequence, thus containing the 3′ junction of IStrons. As a result, the 5′ junction sequence simultaneously satisfies the requirements of (1) the splicing donor of the group I intron, (2) the 5′ junction of the transposon recognized by TnpA, and (3) the TAM for cleavage by TnpB-ωRNA. The 3′ junction functions as (1) the splicing acceptor of the group I intron, (2) the 3′ junction of the transposon recognized by TnpA, and (3) the ωRNA bound to TnpB that precisely dictates the cleavage site.

One striking feature of IStrons is the mutually exclusive functions of RNA, which works as either a self-splicing ribozyme or an ωRNA [84]. IStrons have evolved a sensitive equilibrium to balance competing and mutually exclusive activities within the same transposon-encoded transcript. The presence of TnpA appears to be linked to low ωRNA abundance. Without TnpA, IStrons cannot actively transpose or produce an empty site. TnpB-ωRNA contributes to transposon restorative homing only when mobilized *in trans*. If TnpB-ωRNA still retains endonuclease activity, IStrons without TnpA can propagate by intron homing.

### 5.5. Fanzors—Eukaryotic TnpB Homologs Associated with Various DNA Transposons

TnpB homologs are widely observed in three domains of life [80,81,93]. Eukaryotic TnpB homologs, designated Fanzor, are encoded inside different types of DNA transposons (*Mariner*, *Sola2*, *MuDR*, *Harbinger*, *Helitron*, and *Crypton*) in eukaryotes [80]. Additional cases are reported in Repbase [93,94]: *hAT*, *EnSpm*, *Mariner*, *piggyBac*, *Helitron*, and *Crypton*.

Some Fanzor sequences (Fanzor2) are phylogenetically more closely related to TnpB from the bacterial IS*607* family than other eukaryotic Fanzors (Fanzor1) [80]. Consistently, Fanzor2 is often associated with a serine recombinase gene [80,81]. Here they are designated as *IS607EU* for eukaryotic homologs of IS*607*. It is suggested that Fanzor2 originated from a lineage of TnpB found in cyanobacteria as a part of IS*607*-type DNA transposons [81]. Yet some Fanzor2s are observed inside non-IS*607*-type DNA transposons with TIRs.

Both Fanzor groups are confirmed to be RNA-guided DNA endonucleases [53,93]. In Fanzor (both Fanzor1 and Fanzor2), the catalytic E residue in the RuvC-II subdomain is located just six residues upstream of the zinc finger motif, whereas in canonical prokaryotic TnpB, the catalytic E is around 50 residues upstream of the zinc finger motif [81,93,95]. The same alteration is also observed in some prokaryotic TnpB lineages (designated TnpB2, pro-Fanzor, or RIIr-1 to RIIr-7). It remains undetermined whether it reflects the single origin of Fanzor1 and Fanzor2 along with some prokaryotic TnpB lineages.

Both Fanzor1 and Fanzor2 are also associated with their 3′ transcript, which extends to a 14–15 nt sequence that is not conserved among copies, supporting the similar architecture among Fanzor and TnpB [53,93]. Fanzor1 recognizes 4–5 nt AT-rich TAMs, while Fanzor2 recognizes 3 nt G-rich TAMs [50,53,93,96]. This is consistent with Fanzor2 showing more limited interactions upstream of TAMs.

### 5.6. The OMEGA System as a Composite Homing Endonuclease

There are several lines of experimental support that indicate that the OMEGA system is a composite HE working for transposon restorative homing: (1) They are sequence-specific endonucleases that can recognize a long DNA stretch: TnpB in IS*605* [49,71], IscB in IS*605* [49,74], IsrB in IS*605* [49], TnpB in IS*607* [85], Fanzor1 [53,93], Fanzor2 [53,93]. (2) They can cleave the empty site that is generated by transposon excision and enhance gene conversion to restore the original transposon insertion (restorative homing): TnpB in IS*605* [71,88], IscB in IS*605* [88], TnpB in IS*607* [84]. (3) In addition, there are indications that the OMEGA system acts in other types of homing: TnpB in IStrons is maintained as intact even after the loss of TnpA, implying intron homing [92]. (4) TnpB in IS*605* works as a gene drive agent in a plasmid population [89].

### 5.7. The Possible Mechanisms Maintaining the OMEGA System Without Homing

When the OMEGA system is treated as a selfish entity, what happens if it loses its potential to function as a homing endonuclease? The possibility of the OMEGA system proliferating as a toxin–antitoxin has been proposed [95]. In one proposed system, the TnpB-ωRNA complex acts as a toxin to cleave the target DNA, while the associated DNA-binding protein binds the complex to inhibit DNA cleavage as an antitoxin.

Another likely fate is domestication. Independent incorporation of TnpB into the CRISPR-Cas system as Cas12 is often associated with protein elongation [95]. The functional split of ωRNA into a trans-activating CRISPR RNA (tracrRNA) and a CRISPR RNA (crRNA) is a key process, along with the transition from TnpB to Cas12 [97]. The intermediate TnpB, designated TranC (transposon–CRISPR intermediate), evolved from the TnpB of either the IS*605* or IS*607* family independently multiple times. Most Cas12 subtypes retain nuclease activity, but Cas12c and Cas12m have lost the ability to cleave DNA and instead repress transcription [98,99].

“TnpB-like nuclease-dead repressors (TldRs)” is a recently characterized type of domesticated genes that originated from TnpB [100]. TldRs function with adjacently encoded non-coding guide RNAs to target complementary DNA sequences flanked by a TAM within promoter regions, and target binding downregulates gene expression through competitive exclusion of RNA polymerase.

## 6. Implication of Transposon Restorative Homing

### 6.1. Comparison Among the Types of Homing

In classical intron (or intein) homing, the HE cleaves the intronless (intron−/HEG−) allele, which is the homologous chromosome of the intron+/HEG+ chromosome (Table 1) [21]. In co-conversion or localized marker exclusion, the HE, encoded by the phages or plasmids inside an intron (intron homing) or as a free-standing gene (intronless homing), cleaves the competitor phages or plasmids coinfected in a cell [34,35,36,37,38]. HEs in intronless homing distinguish target (non-self) from non-target (self) based on the sequence differences in a conserved gene nearby the HEG; otherwise, it cleaves the self allele. HEGs can spread via intron homing within the same population or related populations or species. HEGs can spread via intronless homing into related populations or species, but not (or only ineffectively) within the same population.

In transposon restorative homing, the HE cleaves the chromatid from which the transposon is excised after replication, and the cleaved chromatid is repaired by gene conversion with its sister chromatid that retains the transposon insertion [71,88]. This happens because sister chromatids are identical when replicated, and the transposon is excised only from one chromatid. HEs in transposon homing distinguish targets from non-targets based on the absence of a transposon, while HEs in intron homing distinguish targets from non-targets based on the absence of an intron. DNA transposons are excised at the DNA level, while self-splicing introns are excised (spliced) at the RNA level. Restorative homing only works for transposons that are excised at the DNA level. Restorative homing cannot directly contribute to the spread of HEGs, but it can do so through the replication of the transposon encoding the HEG.

Theoretically, the transposon can also be copied after cleavage of the orthologous site in homologous DNA (chromosomes, phages, or plasmids), similar to intron homing and intronless homing. The spread of the IS*605* insertion in a plasmid population is facilitated by TnpB activity, but is not dependent on TnpA activity [89]. The spread of IS*605* via restorative homing needs the activity of both TnpA and TnpB. The result is better explained by transposon non-restorative homing, in which the HE cleaves the orthologous site of a plasmid lacking an IS*605* insertion, and the DNA break is repaired using the plasmid retaining the IS*605* insertion as the template.

### 6.2. Transposons Suitable for Transposon Restorative Homing

The model of transposon restorative homing (Figure 4) implies that it is beneficial only for transposons that excise themselves. Although *Gypsy* (*Ty3*/*Gypsy*) LTR retrotransposons, *Copia* (*Ty1*/*Copia*) LTR retrotransposons, and long interspersed elements (LINEs or non-LTR retrotransposons) are observed nearby Fanzor sequences [93], there is no evidence for retrotransposons encoding a Fanzor. As retrotransposons never excise themselves, there seems to be no evolutionary advantage for retrotransposons to retain a Fanzor.

The most abundant type of transposase in nature is DDE transposases [101]. The majority of DDE transposases catalyze cut-and-paste transposition, which must excise transposons from flanking genomic DNA and often generate a double-strand break at the excised site [4]. It resembles the proposed state after the cleavage by an HE in restorative homing. It is one explanation for the relatively low association between the OMEGA system and DDE transposases. Retaining the large OMEGA system might not provide a strong benefit to transposons with a DDE transposase. The stable association of the OMEGA system with either an HUH nuclease (IS*605*) or serine recombinase (IS*607*) in prokaryotes agrees with their scarless excision [84,88]. Yet, some groups of prokaryotic TnpB appear to be encoded within the non-IS*605*/IS*607* family of ISs. TnpB encoded with a DDE transposase (IS*L2*, IS*630*, IS*1031*), tyrosine recombinase, or Cas1 integrase surrounded by TIRs was identified [95]. Therefore, the OMEGA system is likely able to contribute to the evolutionary success of transposons with a DDE transposase.

In eukaryotes, Fanzor, especially Fanzor1, is incorporated in various DNA transposons, including *Helitron* and *Crypton* [80,93], in contrast to the prokaryotic OMEGA system. *Helitron* encodes an HUH nuclease for the catalysis of single-stranded transposition, but *Helitron* conserves two Y residues downstream of the HUH motif, distinct from the single conserved Y residue observed in the IS*200*/IS*605* family [102]. *Helitron* transposase only excises one strand of *Helitron*, and reintegrates it at another site [103,104]. Nevertheless, a covalently closed double-stranded circular DNA intermediate is required for transposition. Double-stranded *Helitron* excision has not been observed experimentally, and the original donor site from which one strand is excised can produce another excised circular DNA, suggesting that the majority (if not all) of *Helitron* donor sites are restored after the single-stranded DNA excision in mammalian systems. Nevertheless, the removal of double-stranded *Helitron* DNA from somatic cells has been observed in maize [105]. The presence of Fanzor in some *Helitrons* would suggest distinct fates for the donor site after the single-stranded DNA excision.

*Crypton* is transposed by its encoding tyrosine recombinase [106,107]. The transposition mechanism of *Crypton* has not been demonstrated experimentally. The proposed model assumes the involvement of an extrachromosomal circular double-stranded DNA intermediate. No free double-strand break generated by tyrosine recombinase is expected. Some *Crypton* families show a target sequence preference; many *CryptonS* elements share the pentanucleotide TATGG at both termini, one of which is considered the target and the other the terminus of *CryptonS* elements [107]. *IS607EU* is the only known example of a eukaryotic DNA transposon encoding a serine recombinase for transposition [80,81].

Except for *Helitron*, *Crypton*, and *IS607EU*, all eukaryotic DNA transposons incorporating Fanzor are DNA transposons encoding a DDE transposase [80,93]. DNA transposons with a DDE transposase generate TSDs at both sides of the integrant upon transposition [3]. TSDs are 1 to 10 bp sequences originating from the target genomic sequence. Some transposon superfamilies show very strict, often AT-rich, TSD sequence specificity: TA for IS*630* and *Mariner*, and TTAA for *piggyBac* and *Kolobok*. TSD sequences can be divergent in many superfamilies such as *MuDR* and *hAT*. The OMEGA system recognizes TAMs via protein–DNA interactions and the downstream sequence through its complementarity to guide RNA [48,85,87]. The 5′ TSD is recognized as a part of the TAM by the REC and WED domains [50,53,81]. A part of the WED domain shows substantial diversity among Fanzor1s, which may evolve to enable recognition of diverse DNA sequences. The 3′ TSD sequence is included in the ωRNA scaffold in the reported structures of Fanzor1 and Fanzor2; therefore, it would not contribute to the target recognition. Unlike Fanzor2 and the prokaryotic OMEGA system, the 3′ end sequence of the ωRNA of Fanzor1 does not form a pseudoknot with a part of the ωRNA scaffold. The inclusion of the 3′ TSD in the ωRNA scaffold along with the loss of the pseudoknot would have given Fanzor1 more flexibility in target choice. The key mechanistic features are preserved during the evolution of Fanzors, but protein elongation distinguishes them from their prokaryotic counterparts in the loading of ωRNA and target DNA. In addition, the DNA unwinding mechanism and R-loop regulation by the REC domain of Fanzor1 may be crucial for its function in the complex chromatin environments of eukaryotes.

Conversely, the rare presence of transposons encoding a DDE transposase and an OMEGA system in prokaryotes might be caused by the less flexible target recognition by the OMEGA system. If so, it reflects the coevolution of the OMEGA system and its associated transposase.

### 6.3. HEGs Suitable for Transposon Restorative Homing

One aspect that cannot be overemphasized is that in transposon restorative homing, the HEG is obligated to change its target sequence specificity every time its host transposon mobilizes. This constraint makes it almost impossible for HEs with protein-based target recognition to evolve. Classical HEs can gradually modify their target DNA sequence specificity through the accumulation of mutations, but cannot change the target to an unrelated sequence [12,13,14]. The only situation in which a classical HE could contribute to the reinstatement of a transposon after excision is that the transposon itself restricts its mobilization into a specific type of sequence, as in the case of the *Dada* superfamily of DNA transposons [108,109]. Each *Dada* family is inserted specifically in repetitive sequences like tRNA, rRNA, or snRNA genes. The phage-encoded free-standing HE SegB is known to specifically cleave tRNA genes [35]. Though it is theoretically possible, no endonuclease related to HEs has been reported in *Dada*.

*KolobokP* is an interesting example of transposons encoding an endonuclease related to protein-based HEs [110]. The proposed role of the ββα-metal endonuclease encoded by *KolobokP* is to enhance unequal crossover between the repeat sequences at both ends of *KolobokP* copies through cleavage, thereby generating *KolobokP* multimers. This model awaits experimental confirmation, but it raises the possibility that HEs contribute functionally to the proliferation of transposons beyond restorative homing.

Base-pairing between the guide RNA and the target DNA would be an ideal mechanism for the frequent, inevitable changes in target specificity. Analogously, DNA can be used as a guide for target cleavage. DNA-guided DNA cleavage activity has been demonstrated in prokaryotic Argonaute homologs (pAgos) [111]. pAgos use a short (13 to 25 nt) 5′-phosphorylated single-stranded guide DNA to cleave a DNA strand complementary to the guide DNA. Many pAgos preferentially accumulate guides derived from SGEs, including plasmids and phages, rather than chromosomal DNA [111,112,113], but they likely load short endogenous DNA fragments that arise during normal DNA metabolism or the degradation of foreign DNA. Because the active generation of DNA fragments from the flanking DNA of transposons can destabilize the region near the transposon, DNA-guided endonucleases like pAgos are less likely candidates for HEs that contribute to transposon fitness. Transcription can produce guide molecules without disrupting the integrity of the DNA surrounding the transposon.

Virtually only RNA-directed endonucleases, such as the OMEGA system, can make the most of the advantages of restorative homing. In other words, restorative homing can be the selective force for the OMEGA system to maintain flexibility in its target sequence specificity.

## 7. Conclusions

In this review, the properties of the OMEGA system were compared with classical HEs. The comparison highlighted the characteristics of the OMEGA system as a composite HE, an SGE that propagates by copying itself at an empty site. It raised the possibility that its flexible target recognition and cleavage, mechanically mediated by base pairing between the target DNA and the guide RNA, have evolved as an adaptation enabling proliferation through transposon restorative homing.

In contrast to classical HEs that recognize target DNA through protein–DNA interactions, the OMEGA system recognizes target DNA through base pairing with guide RNA [49,53,71,74,85,93]. This enables the OMEGA system to change its target DNA sequence every time its host transposon moves, where the surrounding sequences before and after the transposition are entirely different. Transposon restorative homing reinstates the original transposon sequence in a chromatid after excision by gene transfer using the sister chromatid, retaining the intact transposon as a template [71,88]. Beyond transposon restorative homing, the OMEGA system likely spreads effectively into a population, as indicated by the presence of IStrons encoding only TnpB [92] and the spread of IS*605* in a plasmid population [89]. Though awaiting experimental validation, these cases support the general concept of the OMEGA system as a composite homing endonuclease.

The need for frequent target changes, arising from the mechanism of transposon restorative homing, would have been a strong constraint on retaining flexible target recognition in the OMEGA system, while the catalytic mechanism has remained essentially unchanged. The mechanism of TAM recognition has diversified through coevolution with transposons, in which transposon target preference and the TAM preference coordinate. The IS*200*/IS*605* family and the IS*607* family of ISs show distinct 5′ target preferences, and their associated OMEGA systems show different TAM sequence preferences that match the preferences of the host transposons [48,85,87]. These different TAM preferences are accomplished by distinct protein–ωRNA interactions. IStrons could be an extreme case of coevolution, where three distinct components collaborate [92]. The unique property of Fanzor1, particularly its ωRNA structure that dictates the target DNA, might be an adaptation for associating various transposons with different target preferences and TSD lengths [50,53,81]. Bioinformatics studies have revealed associations between the OMEGA system and many types of genes [95]. Some represent domesticated forms of the OMEGA system, such as Cas12 [95,97] and TldRs [100]. Many others are likely OMEGA system components incorporated into DNA transposons. Mechanistic and structural characterization of different OMEGA systems, especially eukaryotic Fanzor proteins and prokaryotic OMEGA systems not associated with either the IS*200*/IS*605* or IS*607* family, would not only provide tools for genome manipulation with diverse TAM preferences, but also contribute to our understanding of the evolutionary constraints of the OMEGA system.

One fundamental question that arose regarding the concept of the OMEGA system as an HE is how it came to be in the first place. Although much more compact than Cas9 and Cas12, the OMEGA system is still more complex than canonical, protein-based HEs, which are often shorter than 300 residues [8,9,10]. One can assume that a protein-based RuvC-like HE preexisted in a transposon and later acquired RNA-based target recognition. One can also expect the incorporation of an ancestral OMEGA system by a transposon. The structural comparison between the ancestral RuvC nuclease and the effector proteins of the OMEGA system revealed gains and losses of protein domains that interact with ωRNA [52]. The comparison also indicated that the new protein domains have replaced the structural functions of ωRNA during the evolutionary path from the OMEGA system to Cas9. The structural evolution of the ωRNA might be the key to understanding the origin of the OMEGA system.

## Figures and Tables

**Table 1:** Comparison of the different types of homing.

Homing Type	Recognized Difference ^1^	Target
Intron homing	+/− intron	Homologous chromosome or coinfected phages/plasmids
Intronless homing	Sequence substitutions	Coinfected phages/plasmids
Transposon restorative homing	+/− transposon	Sister chromatid
Transposon non-restorative homing	+/− transposon	Homologous chromosome or coinfected phages/plasmids

^1^ +/− indicates that HEs distinguish the targets based on the presence or absence of an intron or a transposon.

## Data Availability

No new data were created or analyzed in this study. Data sharing is not applicable to this article.

## Abbreviations

The following abbreviations are used in this manuscript:

Cas	CRISPR-associated
CRISPR	clustered regularly interspaced short palindromic repeat
crRNA	CRISPR RNA
HE	homing endonuclease
HEARO	HNH endonuclease associated RNA and ORF
HEG	homing endonuclease gene
IS	insertion sequence
ISC	Insertion Sequence encoding Cas9 homolog
LTR	long terminal repeat
OMEGA	Obligate Mobile Element-Guided Activity
pAgo	prokaryotic Argonaute homolog
PAM	protospacer adjacent motif
RAGATH	RNAs Associated with Genes Associated with Twister and Hammerhead
reRNA	right end RNA
RM	restriction-modification
SGE	selfish genetic element
TA	toxin–antitoxin
TAM	transposon-associated motif
TE	transposable element
TIR	terminal inverted repeat
TldR	TnpB-like nuclease-dead repressor
tracrRNA	trans-activating CRISPR RNA
TranC	transposon–CRISPR intermediate
TSD	target site duplication
